# Isolation of *Tulasnella* spp. from Cultivated *Paphiopedilum* Orchids and Screening of Germination-Enhancing Fungi

**DOI:** 10.3390/jof9060597

**Published:** 2023-05-23

**Authors:** Na Yao, Baoqiang Zheng, Tao Wang, Xiaolu Cao

**Affiliations:** 1 State Key Laboratory of Tree Genetics and Breeding, Key Laboratory of Tree Breeding and Cultivation of the National Forestry and Grassland Administration, Research Institute of Forestry, Chinese Academy of Forestry, Beijing 100091, China; nayao@caf.ac.cn (N.Y.); zhengbaoqiang@aliyun.com (B.Z.); 2 Beijing Laboratory of Urban and Rural Ecological Environment, Beijing Floriculture Engineering Technology Research Centre, China National Botanical Garden (North Garden), Beijing 100093, China; wangtao@chnbg.cn

**Keywords:** orchid mycorrhizal fungi, symbiosis, Orchidaceae, conservation

## Abstract

Ex situ conservation, an important way to increase the survival and sustainability of endangered species, is widely used in the conservation of endangered orchids. However, long-term ex situ conservation might affect the dominant group of orchid symbiotic fungi, which are crucial for orchid growth and reintroduction. This study investigated the culturable *Tulasnella* spp. associated with *Paphiopedilum* orchids after long-term greenhouse cultivation, and identified germination-enhancing isolates. A total of 44 *Tulasnella* isolates were obtained from the roots of 14 *Paphiopedilum* spp., and 29 of them were selected for phylogenetic analysis. They clustered mainly with *Tulasnella deliquescens*, *Tulasnella calospora*, *Tulasnella bifrons,* and *Tulasnella irregularis*, but included two potential new groups. Compared with published uncultured data, most of the isolates were grouped together with the reported types, and the dominant *Tulasnella* associated with *P. armeniacum* and *P. micranthum* could still be isolated after ten years of cultivation, most of which were the first isolation. In vitro symbiotic germination showed that certain root isolates could promote seed germination (e.g., parm152 isolated from *P. armeniacum*, Php12 from *P. hirsutissimum*, and prhi68 from *P. rhizomatosum*). These data indicated that the dominant *Tulasnella* types colonizing the roots of cultivated *Paphiopedilum* are stable over time, and germination-enhancing fungi colonizing the roots would benefit for seed reproduction after population reintroduction into the wild.

## 1. Introduction

**Figure 1:**
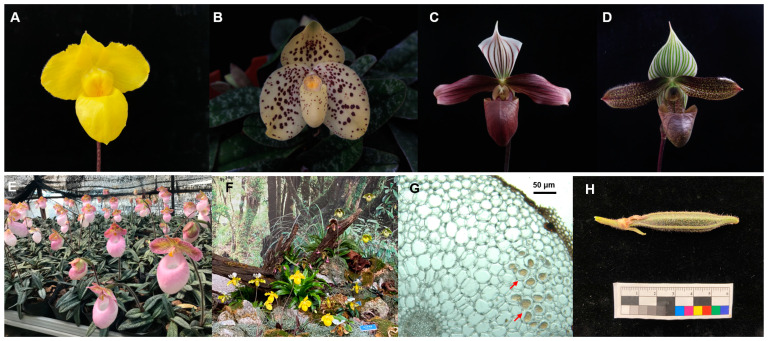
Cultivated *Paphiopedilum* spp. (**A**) *P. armeniacum*. (**B**) *P. wenshanense*. (**C**) *P. purpuratum*. (**D**) *P. wardii*. (**E**) *P. micranthum* cultivated in Xingyi greenhouse. (**F**) Landscape display arranged with *Paphiopedilum* spp. (**G**) Intracellular fungal pelotons in the root of *Paphiopedilum* sp. The red arrows indicate the intracellular fungal pelotons. (**H**) Fruit of *P. hirsutissimum*.

*Paphiopedilum* Pfitzer (Orchidaceae), comprising about 96–100 species around the globe, is mainly distributed in Southeast Asia [1,2] and is popular in the horticultural market and exhibitions as a pot plant because of its slipper-like flower shape and abundant color (Figure 1A–D,F). China is one of the distribution centers of *Paphiopedilum*. There are about 27 native species found in China, most of which are distributed in Yunnan or Guangxi province [3]. Over-collection and loss of suitable habitats have led to the wild population of *Paphiopedilum* being under the threat of extinction [4]. All species in *Paphiopedilum* are listed in the Convention on International Trade in Endangered Species of Wild Fauna and Flora (CITES) [5].

To conserve and restore the population of *Paphiopedilum*, studies of population geographical distribution [6], embryo development [7,8], in vitro tissue culture [9,10], ex situ seed baiting [11], and mycorrhizal fungi associated with *Paphiopedilum* [12] have been carried out. At the same time, in situ/ex situ conservation of *Paphiopedilum* orchids has also been implemented [3]. The limited distribution and endangered status [13] of *Paphiopedilum* means that it has only been sporadically studied compared with other commercial orchids.

Like other orchids, *Paphiopedilum* spp. depend on mycorrhizal fungi from seed germination to seedling growth [14], to obtain organic nutrients and carbon energy. Orchid mycorrhizal fungi (OMF) form characteristic intracellular hyphae (Figure 1G), called a peloton, within the cells of *Paphiopedilum* orchid roots or protocorms [15]. Most of the OMF associated with *Paphiopedilum* orchids are members of the genus *Tulasnella* [12,16,17] and *Ceratobasidium* [4,15].

*Tulasnella* J. Schröt. (Tulasnellaceae) is an important genus of OMF that occurs worldwide in the form of mycobionts associated with orchids [18,19,20], which is also a frequently detected fungal group in the roots of *Paphiopedilum* [21]. Diverse *Tulasnella* fungi colonize *Paphiopedilum*. In southwestern China, Yuan et al. [12] amplified 25 types of internal transcribed spacer (ITS) sequence representing Tulasnellaceae from the roots of *Paphiopedilum* and *Cypripedium*. Similarly, *Tulasnella* species such as *T. calospora*, *T. violea*, and *T. pruinosa* were isolated from five species of *Paphiopedilum* in Thailand [16].

To date, most species in *Paphiopedilum* have been successfully cultivated in the greenhouse for ex situ conservation. In the present study, we isolated *Tulasnella* spp. from 14 *Paphiopedilum* species cultivated in greenhouses in southwestern China, and used phylogenetic analysis of ITS sequences to identify and characterize the isolates. We focused on the following issues: (1) After long-term cultivation, do *Tulasnella* spp. exist in specific *Paphiopedilum* species? (2) Do *Tulasnella* spp. isolated from cultivated *Paphiopedilum* have a positive effect in promoting seed germination? These issues are very important for seedling adaptability and seed germination of *Paphiopedilum* population reintroduction into the wild.

## 2. Materials and Methods

### 2.1. Study Materials

*Paphiopedilum* species, including *P. armeniacum* (Figure 1A), *P. callosum*, *P. emersonii*, *P. gratrixianum*, *P. henryanum*, *P. hirsutissimum*, *P. malipoense*, *P. micranthum* (Figure 1E), *P. purpuratum* (Figure 1C), *P. tigrinum*, *P. villosum*, *P. wardii* (Figure 1D), *P. wenshanense* (Figure 1B), and *P. rhizomatosum*, were ex situ conserved in a greenhouse in Xingyi, Guizhou province, southwestern China. The orchids were cultivated on pine bark substrate in the greenhouse for at least 3 years before root sampling. The seedlings were healthy, and some were at the flowering stage or fruiting stage after artificial pollination. The root samples were collected from September 2017 to May 2021 (Table S1). *Paphiopedilum* spp. at different growth stages were randomly selected for sampling, with three plants for each stage, and 2–3 healthy roots from each plant. Sampled seedlings were marked to avoid subsequent sampling. About 5–10 cm of the healthy root was cut from each seedling using a sterile scalpel, put into a sterile bag, and transported at 4 °C for 24 h until fungal isolation.

The fruit of *P. hirsutissimum* were obtained by artificial pollination (Figure 1H). Seeds at approximately 130–160 days after pollination were used for in vitro germination. Healthy uncracked capsules were harvested from 2019 to 2021, which were cultivated in the greenhouse of Chinese Academy of Forestry, Beijing, China.

### 2.2. Fungal Isolation and Identification

The roots were transferred to the laboratory, washed using sterile water, and surface sterilized (30 s submergence in 75% ethanol, 30 s rinsing with sterile distilled water, 5 min submergence in 1% sodium hypochlorite, and 30 s rinsing in sterile distilled water five times). The sterilized roots were sliced into 0.5–1.0 mm sections and inoculated on one-quarter strength potato dextrose agar (PDA) from BD Difco (Franklin Lakes, NJ, USA) with 11.25 g/L agar at 25 ± 2 °C. Five sections were placed in one Petri dish for fungal isolation, with 10 dishes per sample. When hyphae growing from the section exceeded 0.5 cm in length, the tips of the hyphae were cut and transferred to new one-quarter strength PDA medium for purification. After repeating this purification step four to five times, purified isolates were obtained.

The purified isolates were identified morphologically by observing and recording the color and surface shape of their culture characteristics [22,23,24] on PDA medium at 25 °C in the dark for about 1–4 weeks, depending on their growth rates. The condition of their nuclei was observed from young hyphae after staining with SYBR Green I according to a previous publication [25]. Observation, measurement, and photography of microscopic fungal structures were recorded using a ZEISS Axio Imager M2 microscope (ZEISS, Oberkochen, Germany), with a ZEISS Axiocam 503 mono camera and differential interference contrast (DIC) illumination. ZEISS Zen 3.3 was used for image processing and synthesis.

For molecular identification of the isolates, the ribosomal DNA (rDNA) region containing the two ITS regions and the 5.8S gene was amplified using primers ITS1 and ITS4 [26]. The PCR was performed as previously described [27]. The PCR products were purified and sequenced at Sangon Biotech Co., Ltd. (Beijing, China) using the same primers. The sequences were BLAST searched against the GenBank database of the National Center for Biotechnology Information (NCBI) for identification. The sequences were then deposited in the GenBank database (Table S1). The isolates were maintained at the Research Institute of Forest, Chinese Academy of Forestry.

### 2.3. Phylogenetic Analysis of the ITS Sequences

As previously noted, within *Tulasnella*, the ITS sequences could reveal congruent species delimitation and phylogenetic outcomes [28]. The sequences representing the two ITS regions and the 5.8S gene were used for the phylogenetic analysis of *Tulasnella*. One phylogenetic tree was constructed by combining the ITS sequence of the isolates with the published ITS sequences of *Tulasnella* spp. downloaded from GenBank in NCBI (Table S2) to determine the taxonomic status of the isolates [20,29]. Another phylogenetic tree was analyzed to clarify the relationship among the isolates associated with *Paphiopedilum* spp. The uncultured ITS sequences of Tulasnellaceae amplified from roots of *Paphiopedilum* spp. and *Cypripedium* spp. in a previous study [12] were used as reference sequences to investigate the continuous colonization of *Tulasnella* associated with *Paphiopedilum* spp.

All the sequences used for phylogenetic analyses were aligned using MAFFT v7.311 [30] and manually adjusted using MEGA 7 [31]. MEGA 7 was used to construct the maximum likelihood (ML) trees under the general time reversible model (GTR+I+G), with 1000 bootstrap replicates. MrModeltest v2.3 [32,33] was used to determine the best-fit evolution model for the combined dataset for Bayesian inference (BI). BI was calculated using MrBayes v3.2.7 [34], with a GTR+I+G model of DNA substitution and a gamma distribution rate variation across sites. Four Markov chains were run for two runs from random starting trees for 1 million generations and were sampled every 100 generations. The phylogenetic trees were visualized using FigTree v1.4.2 (http://tree.bio.ed.ac.uk/software/figtree/ (accessed on 16 November 2021)). Branches that received bootstrap support for ML and Bayesian posterior probabilities greater than or equal to 50% and 0.70, respectively, were considered to be significantly supported.

### 2.4. Screening of Germination-Enhancing Fungi

Fungi from different clades in the phylogenetic tree were tested for their ability to enhance the germination of *P. hirsutissimum* seeds. After surface sterilization of the capsules as described by Zi et al. [35], approximately 200–300 seeds were sown on oatmeal agar (OMA) medium (3.0 g of oatmeal (Solarbio, Beijing, China) was added to 800 mL of distilled water, boiled for 30 min, filtered through a 50-mesh sieve to remove large particles, added 6.0 g of agar, and then added to 1000 mL with water ) in a Petri dish. A fungal inoculum was placed in the center of each dish and co-inoculated with the seeds. Asymbiotic germination was performed as a control on OMA or MS1 (MS medium with one-quarter strength MS macronutrients; 0.4 mg/L 6-benzylaminopurine, 10.0 g/L sucrose, 6.0 g/L agar, pH 6.3). There were three replicates for each treatment. All replicates were placed in germination chambers at 25 ± 2 °C and subjected to alternating 12 h periods of light and dark. Three independent sowing experiments were performed.

**Figure 2:**
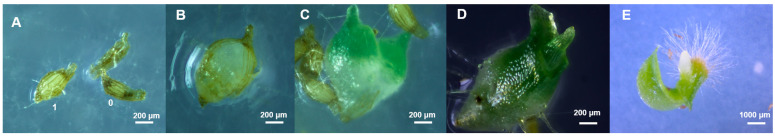
Seed germination stages of *P. hirsutissimum.* (**A**) 0. Seed of *P. hirsutissimum*, ungerminated; 1. Embryo swells and testa are propped up (germinated). (**B**) Embryo enlargement results in a spherule, and the seed coat is broken (protocorm formation). (**C**) Appearance of the protomeristem (protocorm differentiation). (**D**) A seedling with leaf emergence. (**E**) A seedling with root emergence.

The number of seeds and the germination status in each dish were assessed after 90 days of incubation, according to previously defined stages (Figure 2) [36]. The percentages of germinated seeds at each developmental stage were calculated. Seed germination data were recorded as previously described [37]: 0, 1, (2 + 3), and (4 + 5) stages were defined based on the number of ungerminated, germinated (g) seeds, protocorms (p), and seedlings (s). The total number of seeds (t) constituted the seeds with well-developed embryos. Germination rate (G), protocorm formation rate (P), and seedling formation rate (S) were calculated 90 days after inoculation. The following calculations were used:

G = 100 × (g + p + s)/t.(1)

P = 100 × p/t.(2)

S = 100 × s/t.(3)

Analysis of variance was performed using SPSS 16.0 (IBM Corp., Armonk, NY, USA). The data were analyzed using one-way analysis of variance (ANOVA) after inverse sine transformation. Statistical significance was set at *p* < 0.05.

## 3. Results

### 3.1. Fungal Isolation and Morphological Characterization

A total of 14 *Paphiopedilum* species cultivated in a Xingyi greenhouse were sampled at different growing stages, including the vegetative growth, flowering, or fruiting stage. In total, 44 fungal isolates belonging to *Tulasnella* were obtained, which were identified by BLAST analysis of the ITS sequences. For the closest matching ITS sequence, 25 isolates were BLAST searched against uncultured Tulasnellaceae clones. For the source of the closest matching fungi, 28 isolates were BLAST searched against *Paphiopedilum* spp., and 2 isolates were BLAST searched against *Phragmipedium longifolium* (Table S1), which is a member of the subfamily Cypripedioideae.

**Figure 3:**
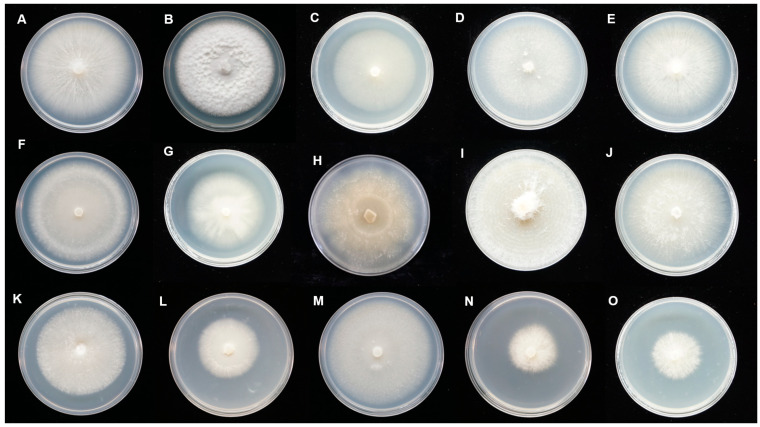

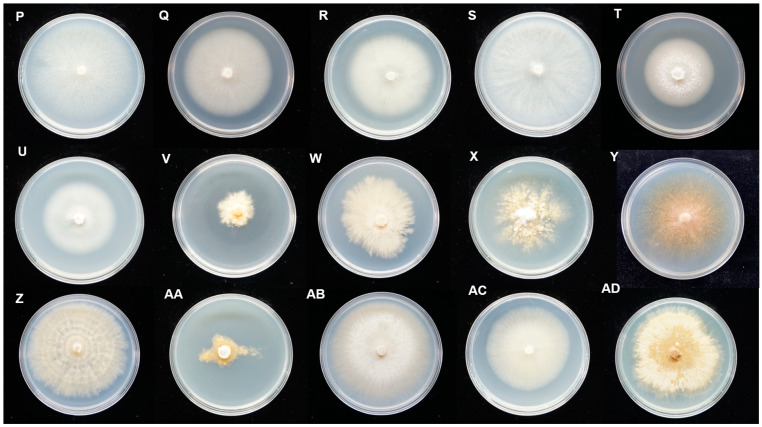
The colonial morphological characteristics of representative *Tulasnella* spp. isolated from *Paphiopedilum* cultured on potato dextrose agar (PDA). Group A: (**A**) parm152; (**B**) parm95; (**C**) parmf61; (**D**) parm451; (**E**) parm712; (**F**) Php18; (**G**) pemer202; (**H**) pgrat15; (**I**) pgrat61; (**J**) phir882; (**K**) Php12; (**L**) Php25; (**M**) pmi16; (**N**) pmilg281; (**O**) pmir712; (**P**) pmir45; (**Q**) prhi65; (**R**) ptigf131; (**S**) pbs1191; (**T**) pwar310; (**U**) pwar205. Group B: (**V**) parm31; (**W**) parm382; (**X**) parmf59; (**Y**) pgrat632; (**Z**) pjack49; (**AA**) ppur922; (**AB**) prhi68; (**AC**) pwen31; (**AD**) parm11.

**Figure 4:**
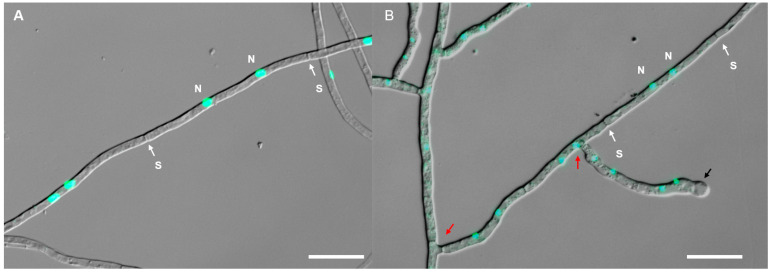
Hyphae of *Tulasnella* spp. stained with SYBR Green I showing binucleate cells. (**A**) Php12; (**B**) pjack49 (N = nuclei; S = septa; the red arrows indicate hyphae with branching at right angles; the black arrow indicates a monilioid cell chain). Bars = 20 µm.

These isolates showed diverse colonial morphology on PDA medium (Figure 3), and could be divided into two groups. In Group A, the colonies were white or beige with aerial and submerged hyphae. In Group B, the colonies were light buff to orange with submerged hyphae. The hyphae were regularly septate with branching at right angles, hyaline, and with binucleate cells (Figure 4). Molinioid cells were hyaline, barrel to elliptical-shaped, and occurred in branched chains (Figure 4B). Sexual morphology was not observed for all the isolates. Combined with the BLAST results and colonial characteristics, 29 representative isolates (Figure 3), removing the isolates that were repeatedly acquired at each isolation, were finally selected for the subsequent phylogenetic analysis (Table 1).

### 3.2. Phylogenetic Analysis of the Representative Isolates

**Figure 5:**
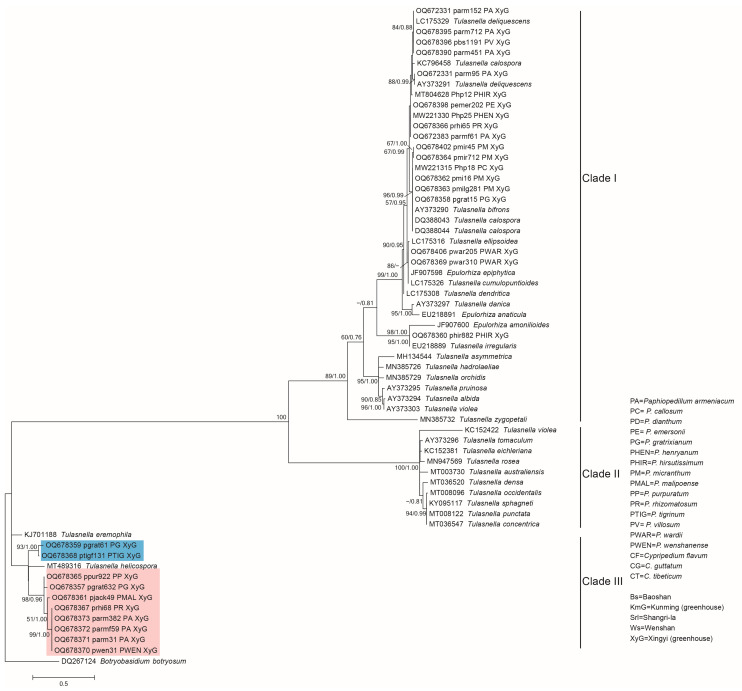
Maximum likelihood phylogenetic tree for *Tulasnella* based on internal transcribed spacer (ITS) sequence alignment. Maximum likelihood bootstrap support (ML > 50) and Bayesian posterior probability (PP > 0.70) values are indicated next to the nodes (ML/PP). *Botryobasidium botryosum* was used as the outgroup. Color blocks highlight the two potential new groups.

The ITS sequences consisted of 63 isolates (including the outgroup sequence DQ267124 *Botryobasidium botryosum*), of which 34 were published sequences downloaded from NCBI (Table S2) and 29 were the representative isolates from this study (Table 1), which were used for phylogenetic analysis. The phylogenetic tree showed that mycorrhizal fungi isolated from *Paphiopedilum* orchids were in the genus *Tulasnella* (Figure 5), which were mainly clustered with *Tulasnella deliquescens*, *Tulasnella calospora*, *Tulasnella bifrons,* and *Tulasnella irregularis*. There were two potential new groups in Clade III (new group one: pgrat61 and ptigf131; new group two: pgrat632, ppur922, pjack49, prhi68, parmf59, parm382, parm31, and pwen31), which were not clustered with previously reported *Tulasnella* species. This indicated that after long-term cultivation in similar environments, the *Tulasnella* fungi in different *Paphiopedilum* orchids belonged to different species, rather than converging on a few/one species.

In the tree of *Tulasnella*, the 29 isolates fell into two clades (Figure 5). Nineteen isolates were clustered in Clade I, and ten isolates were clustered in Clade III. Meanwhile, the fungi in Clade II were not isolated. The 19 isolates belonging to Clade I were mainly from 10 *Paphiopedilum* species, and the isolates belonging to Clade III were from 7 *Paphiopedilum* species. The roots of *P. armeniacum*, *P. gratrixianum*, and *P. rhizomatosum* were colonized by two types of isolates belonging to Clade I and Clade III.

Some isolates with a similar phylogenetic status were acquired multiple times from one *Paphiopedilum* species at different isolations (Table 1, Figure 5), such as pmi16, pmilg281, pmir45, and pmir712 from *P. micranthum* and parm31, parm382, and parmf59 from *P. armeniacum*, which indicated that some *Tulasnelloid* fungi could stably colonize the roots of *Paphiopedilum* orchids within a few years. Most of the above isolates were isolated for the first time in this study, because the closest sequence alignments in NCBI were from uncultured clones.

**Figure 6:**
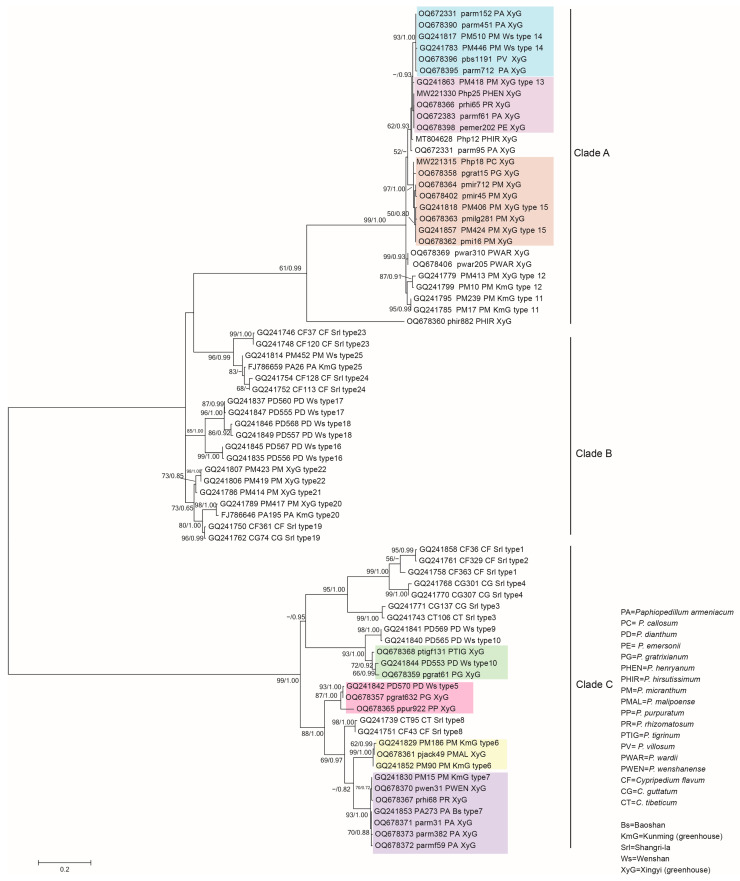
Maximum likelihood phylogenetic tree for mycorrhizal fungi obtained from the subfamily Cypripedioideae based on ITS sequence alignment. Maximum likelihood bootstrap support (ML > 50) and Bayesian posterior probability (PP > 0.70) values are indicated next to the nodes (ML/PP). The sequences with types were Tulasnellaceae sequences obtained and typed by Yuan [12] from the subfamily Cypripedioideae. Color blocks highlight the seven common types.

Another phylogenetic tree (Figure 6) was constructed based on the ITS sequences of the 29 isolates and the 25 types of published Tulasnellaceae sequences amplified from the roots of *Paphiopedilum* and *Cypripedium*. Seventeen of the twenty-five types were from *Paphiopedilum*, and eight types were from *Cypripedium*. The phylogenetic tree showed that 24 isolates in this study were clustered with 7 of the 17 types from *Paphiopedilum*. The eight types from *Cypripedium* were not isolated from *Paphiopedilum*. The isolates parm95, Php12, pwar310, pwar205, and phir882 were new types. As in the *Tulasnella* tree (Figure 5), all the 29 isolates were mainly clustered into two clades. Clade A and Clade C corresponded to Clade I and Clade III in the tree of *Tulasnella*, respectively.

Fourteen isolates were clustered with type 15 and type 13 from the Xingyi greenhouse, and type 14 from Wenshan, which were in Clade A. Clade C included 10 isolates, which were clustered with type 10 and type 5 from Wenshan, type 6 from Kunming, and type 7 from different regions (Kunming and Baoshan).

It should be noted that fungi belonging to type 15 and type 7 could still be isolated after about ten years of cultivation, which indicated that some *Tulasnella* spp. could colonize the roots of *P. micranthum* and *P. armeniacum* for a long time. Meanwhile, some types included isolates from different *Paphiopedilum* species, such as the isolates (parm152, parm415, pbs1191) from *P. armeniacum*, and *P. villosum* clustered into type 14 and isolates (Php18, pgrat15, pmir712) from *P. callosum*, *P. gratrixianum*, and *P. micranthum* clustered into type 15.

### 3.3. Fungal Capacity to Enhance Germination

**Figure 7:**
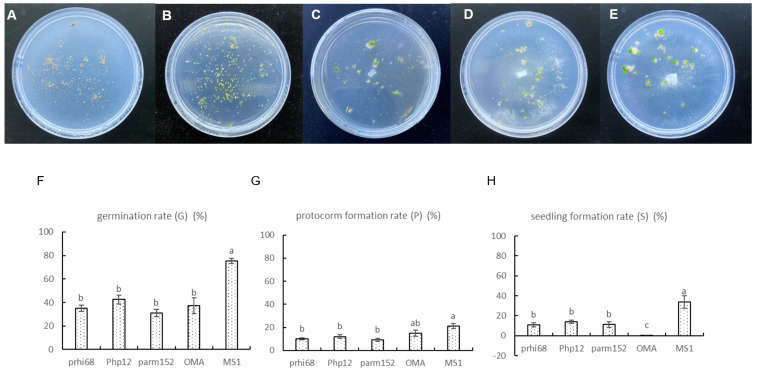
In vitro symbiotic germination of *P. hirsutissimum* with germination-enhancing fungi. (**A**,**B**) In vitro germination of seeds on oatmeal agar (OMA) (**A**) and MS1 (**B**) media in 90 mm Petri dishes. (**C**–**E**) In vitro symbiotic germination of seeds with fungal isolates prhi68 isolated from *P. rhizomatosum*, Php12 from *P. hirsutissimum*, and parm152 from *P. armeniacum* in 90 mm Petri dishes. (**F**–**H**) Rates of seed germination, protocorm formation, and seedling development after 90 days of germination. Different lowercase letters are significantly different (*p* ˂ 0.05).

After 90 days of in vitro symbiotic germination on OMA medium, three isolates could significantly promote the seedling formation of *P. hirsutissimum* (Figure 7C–E) compared with that on the asymbiotic negative control (OMA) (Figure 7A). The in vitro symbiotic germinated protocorms were checked microscopically for mycorrhizal colonization. The three isolates could colonize protocorm cells of *P. hirsutissimum* and formed the typical intracellular hyphae coils of OMF (Figure S1).

The asymbiotic positive control (MS1) allowed more than 75% seed germination (Figure 7F), leading to large proportions of seedlings (33.98%; Figure 7B). In the asymbiotic negative control treatment (OMA), seedling formation was difficult. With the extension of incubation time, most of the protocorms and germinated seeds on OMA at stage 1–3 gradually became brown and died instead of developing into seedlings. Meanwhile, during symbiotic germination, about 10% of healthy seeds could develop into seedlings (Figure 7H).

Three germination-enhancing fungi (prhi68, Php12, and parm152) were isolated from three different *Paphiopedilum* species: *P. rhizomatosum*, *P. hirsutissimum*, and *P. armeniacum*. Isolate prhi68 belonged to Clade III/Clade C in the phylogenetic trees, which was a potential new taxon. Isolate Php12, as a native fungus for the seeds of *P. hirsutissimum*, belonged to a new type in Clade A (Figure 6). Isolate parm152 belonged to type 14 reported previously. The three isolates had similar efficiencies in promoting protocorm and seedling formation for *P. hirsutissimum*, which were significantly higher than in the asymbiotic negative control treatment (OMA) (Figure 7G,H). The seedling morphologies after symbiotic germination with the three fungi were different. Isolate parm152 promoted more obvious root development.

## 4. Discussion

*Tulasnella* spp. are among the most common OMF found in the roots of orchids [38]. The main results in this study indicated that long-term greenhouse cultivation might not lead to a complete change of the dominant group of *Tulasnella* associated with *Paphiopedilum* spp. At the same time, we identified germination-enhancing isolates among the fungi isolated from the roots of *Paphiopedilum* spp., indicating the potential for reintroduction and seed reproduction of ex situ conserved *Paphiopedilum* spp., which could release the pressure of population extinction for orchids in China [39].

### 4.1. Fungal Isolates Associated with Paphiopedilum

The *Paphiopedilum* spp. in this study have been raised in the greenhouse for more than 3 years; therefore, we speculated that under long-term cultivation, the *Tulasnella* spp. in the Xingyi greenhouse would tend to lose their diversity [40]. However, by analyzing the ITS sequences of 29 representative isolates, it was found that they were mainly clustered with four *Tulasnella* species, and two potential new taxa were observed, which indicated that the culturable *Tulasnella* spp. associated with *Paphiopedilum* spp. in the Xingyi greenhouse were certainly diverse.

The isolates in Clade I were represented mainly by *Tulasnella deliquescens* and *Tulasnella calospora*, which are common mycorrhizal fungi found in *Paphiopedilum* species [21]. *Tulasnella calospora* has been proven to have the effect of promoting seed germination and plant growth of many orchid plants, such as *Cymbidium* [41], *Dendrobium* [35], *Paphiopedilum* [14], and *Bletilla* [42], and is also an important species in research on the symbiosis mechanism of OMF [43]. In this study, different isolates were acquired from cultivated *Paphiopedilum* spp., including germination-enhancing isolates, which can provide microbial resources for comparative research on the functional diversity of *Tulasnella* spp. associated with *Paphiopedilum* spp.

The isolates from *P. armeniacum*, *P. gratrixianum*, and *P. rhizomatosum* included both Clade I and Clade III species. *Tulasnella* spp. isolated from *Paphiopedilum* in Thailand also included two clades, which corresponded to Clade I and Clade III in this study [16]. Meanwhile, in most related research, only one clade of *Tulasnella* was isolated. This might have been caused by the unsuitable culture conditions for the growth of some *Tulasnella* fungi. In future research, a culturomics approach for *Tulasnella* isolation could be adopted according to the techniques in environmental microbial culturomics [44].

The isolates in Clade III contained potential new fungal taxa, which were not clustered with previously reported *Tulasnella* species. The best matching fungal ITS sequences close to these isolates were mainly from the orchids in the subfamily Cypripedioideae, such as *P. armeniacum* [12], *P. micranthum* [12], *P. callosum* [16], *P. dianthum* [12], and *Phragmipedium longifolium* (direct submission). Some of the isolates in Clade III were first reported and described in this study because the best matching fungal ITS sequences were obtained through uncultured methods. Strict identification of new fungal taxa requires obtaining more isolates with intraspecific differences [45,46]. In this study, only one new taxon included more than three different isolates. Therefore, currently, they can only be referred to as potential new taxa. In future research, we hope that we can obtain more isolates to determine their taxonomic status.

### 4.2. Continuous Colonization of Tulasnella in Paphiopedilum

Wild populations of *Paphiopedilum* in China have been strictly protected in recent years [47,48]. The *Paphiopedilum* spp. used in this study were collected from the germplasm conservation greenhouse in Xingyi. After long-term greenhouse cultivation, whether *Tulasnella* spp. that once colonized in the roots still exist, and whether these *Tulasnella* spp. could promote seed germination are issues of great concern to us. The diversity of *Tulasnella* in some *Paphiopedilum* species in this greenhouse had been studied using an unculturable method [12]. In this study, the phylogenetic tree indicated that most of the isolates could be grouped together with *Tulasnella* associated with *Paphiopedilum* that were published previously. Especially for *P. armeniacum* and *P. micranthum*, their dominant *Tulasnella* groups were basically consistent with Yuan’s report [12]. This means that some isolates can still colonize *Paphiopedilum* spp. for a long period of time.

We speculated that *Tulasnella* spp. could continue to form symbiosis with *Paphiopedilum*, considering that the geographical location of the Xingyi greenhouse is close to the original habitat of *Paphiopedilum*, and the samples collected in the greenhouse are propagated by division mother plants. The reason for this continuous colonization might be that the cultivation environment, such as the nutrient and water conditions, are close to the wild conditions [49,50,51], or that *Tulasnella* spp. survived in cultivation substrates could be recruited by species of *Paphiopedilum*, or that the propagation method facilitates the colonization of the fungi from mother plants [52], or that like other orchids, *Paphiopedilum* spp. prefer specific fungal groups [53,54,55]. Currently the results of this study are supported by culturable data from isolates, and it is expected that, in future research, amplification sequencing based on high throughput sequencing will be conducted for in-depth analysis [56,57].

### 4.3. In Vitro Symbiotic Germination of Paphiopedilum

In the wild, *Paphiopedilum* seeds germinate relatively slowly because of the seeds’ morphological and physiological characteristics [10,58]. In asymbiotic germination, the germination percentage of many *Paphiopedilum* species is extremely low and is often affected by many factors [9], such as plant hormone content in the embryo [8] or non-methylated lignin accumulation in the seeds [59]. Therefore, advances in hybrid breeding of *Paphiopedilum* and rapid propagation through seed germination is relatively slow.

*Tulasnella* spp. play an important role in enhancing the symbiotic germination of orchid seeds. However, there is a limited number of isolates that can promote the germination of *Paphiopedilum* [11,14,15]. At the beginning of the study, we used the seeds of *P. armeniacum* and *P. micranthum* for in vitro symbiotic germination; however, the germination rates were too low to conduct statistical analyses. After the pre-experiment, the seeds of *P. hirsutissimum* were used to test the capacity of fungi to promote seed germination. Finally, we screened three isolates from different *Paphiopedillum* spp. that could stably promote the germination of *P. hirsutissimum*, which suggested that the specificity of germination enhancement between species of *Tulasnella* and *P. hirsutissimum* was not very strict. Similar results were found for *Cynorkis* [60]. However, this was not consistent with a previous report on fungi isolated from naturally occurring protocorms [11,61], which might be because the fungi isolated from in situ/ex situ protocorms or seedlings were more specific.

Not all the *Tulasnelloid* fungi colonizing roots were active in seed germination or seedling formation [37]. Some orchids might use certain mycorrhizal fungi only in the early stages and they do not necessarily continue to be associated with them in the adult stage [62,63]. The studies on fungal isolation from in situ/ex situ germinated protocorms are gradually increasing, and these fungi are believed to be more specific for seed germination [64,65,66,67]. The germination efficiency of *Paphiopedilum* in the wild is very low; therefore, we used the traditional method to isolate fungi from the root. Many germination-enhancing fungi were acquired by this method in previous reports [68,69]. In future research, we will attempt to conduct ex situ symbiotic germination in the greenhouse to acquire more specific mycorrhizal fungi.

## 5. Conclusions

Based on the published ITS sequences of *Tulasnella* spp. and the 29 representative isolates in this study, we found that after long-term greenhouse cultivation, some *Paphiopedilum* spp. could still maintain colonization of the dominant *Tulasnella* type in their roots. Through in vitro symbiotic germination, germination-enhancing isolates were screened, which indicated that some of the *Paphiopedilum* orchids in the greenhouse had the potential for seed reproduction after reintroduction to the wild. These findings will provide abundant fungal resources for future studies of the mechanism of the specific interaction between *Paphiopedilum* and *Tulasnella*, and the symbiotic germination mechanism. Meanwhile, we could also provide beneficial *Tulasnella* fungi to construct a healthy root fungal community for tissue-cultured seedlings of *Paphiopedilum* in a greenhouse, which is beneficial for the artificial reproduction of *Paphiopedilum*. Furthermore, it will promote the commercial cultivation of *Paphiopedilum* and drive the development of flower planting in remote areas in southwest China.

## Figures and Tables

**Table 1:** The 29 representative *Tulasnella* spp. isolates from *Paphiopedilum*.

No.	Fungal Isolate	Orchid	Accession Number	Closest Match in GenBank	Percentage Identity (%)	Source of Closest Matching Fungi	Culturable
1	parm152	*P. armeniacum*	OQ672311	MN918479	100	NA	Isolate
2	parm31	*P. armeniacum*	OQ678371	GQ241853	99.39	*P. armeniacum*	Uncultured
3	parm382	*P. armeniacum*	OQ678373	GQ241853	98.69	*P. armeniacum*	Uncultured
4	parm451	*P. armeniacum*	OQ678390	GQ241817	99.51	*P. micranthum*	Uncultured
5	parm712	*P. armeniacum*	OQ678395	KF537647	99.01	*Liparis japonica*	Isolate
6	parm95	*P. armeniacum*	OQ672331	JQ247568	100	soil	Isolate
7	parmf59	*P. armeniacum*	OQ678372	GQ241854	100	*P. armeniacum*	Uncultured
8	parmf61	*P. armeniacum*	OQ672383	KX387616	99.6	*P. hirsutissimum*	Uncultured
9	pbs1191	*P. villosum*	OQ678396	GU166415	99.66	*P. charlesworthii*	Isolate
10	pemer202	*P. emersonii*	OQ678398	OP740400	100	*Habenaria longicornu*	Isolate
11	pgrat15	*P. gratrixianum*	OQ678358	EF393621	98.99	*Cymbidium floribundum*	Isolate
12	pgrat61	*P. gratrixianum*	OQ678359	FJ940903	96.64	*Pecteilis susannae*	Isolate
13	pgrat632	*P. gratrixianum*	OQ678357	GQ241842	98.96	*P. dianthum*	Uncultured
14	phir882	*P. hirsutissimum*	OQ678360	GU166423	99.45	*Cymbidium*	Isolate
15	Php12	*P. hirsutissimum*	MT804628	OQ148693	98.94	*Dendrobium flexicaule*	Isolate
16	Php18	*P. callosum*	MW221315	GU166421	99.82	*P. villosum*	Isolate
17	Php25	*P. henryanum*	MW221330	KX387600	99.81	*P. dianthum*	Uncultured
18	pjack49	*P. malipoense*	OQ678361	GQ241829	98.76	*P. micranthum*	Uncultured
19	pmi16	*P. micranthum*	OQ678362	GQ241818	99.82	*P. micranthum*	Uncultured
20	pmilg281	*P. micranthum*	OQ678363	GQ241857	98.05	*P. micranthum*	Uncultured
21	pmir45	*P. micranthum*	OQ678402	GQ241818	97.47	*P. micranthum*	Uncultured
22	pmir712	*P. micranthum*	OQ678364	OL638255	98.15	*Cymbidium goeringii*	Isolate
23	ppur922	*P. purpuratum*	OQ678365	GU166424	97	*P. callosum*	Isolate
24	prhi65	*P. rhizomatosum*	OQ678366	KX387600	99.64	*P. dianthum*	Uncultured
25	prhi68	*P. rhizomatosum*	OQ678367	GQ241830	99.64	*P. micranthum*	Uncultured
26	ptigf131	*P. tigrinum*	OQ678368	KC478574	97.34	*Phragmipedium longifolium*	Uncultured
27	pwar205	*P. wardii*	OQ678406	AJ313448	99.16	*Oncidium*	Isolate
28	pwar310	*P. wardii*	OQ678369	MW432189	99.82	*Dendrobium officinale*	Isolate
29	pwen31	*P. wenshanense*	OQ678370	GQ241830	99.53	*P. micranthum*	Uncultured

NA: no separation information. Source of Closest Matching Fungi: except for special annotations, the fungal sequences are from the roots of orchids.

## Data Availability

The data used in the study are available upon request from the corresponding author.

## Supplementary Materials

The following supporting information can be downloaded at: https://www.mdpi.com/article/10.3390/jof9060597/s1, Figure S1: Fungal hyphae colonizing the inner cells of protocorms; Table S1: Forty-four fungal isolates from roots of *Paphiopedilum*; Table S2: Thirty-three published *Tulasnella* spp.
