# Supplementary material for: Isolation of Tulasnella spp. from Cultivated Paphiopedilum Orchids and Screening of Germination-Enhancing Fungi
Source: J Fungi (Basel). 2023 May 23;9(6):597. doi: 10.3390/jof9060597 (PMC10299374; doi:10.3390/jof9060597)
Supplement: Supplementary file 1 [file jof-09-00597-s001.zip › jof-2389684-supplementary.pdf]

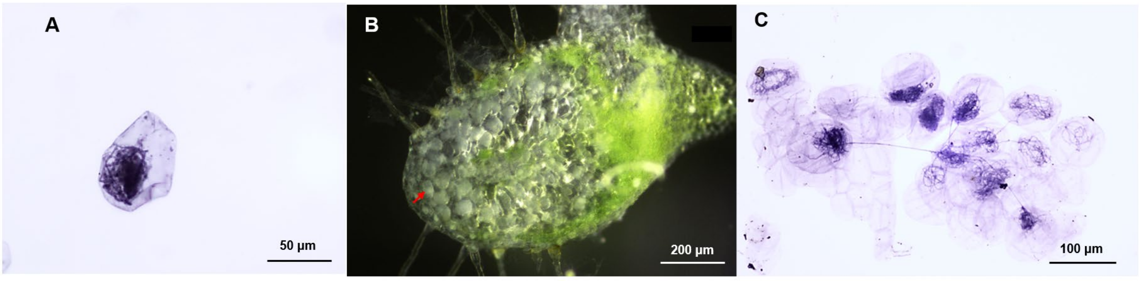

Figure S1 Fungal hyphae colonizing in the inner cells of protocorms and formed the intracellular hyphae coils after *in vitro* symbiotic germination with (A) prhi68, (B) Php12, (C) parm152. The red arrow indicates the intracellular fungal peloton.

The hyphal coils staining method was described in reference 42.
The cells and protocorms were observed under BX41 microscope (Olympus, Tokyo, Japan).
